# Design and rationale of a prospective, collaborative meta-analysis of all randomized controlled trials of angiotensin receptor antagonists in Marfan syndrome, based on individual patient data: A report from the Marfan Treatment Trialists' Collaboration

**DOI:** 10.1016/j.ahj.2015.01.011

**Published:** 2015-05

**Authors:** Alex Pitcher, Jonathan Emberson, Ronald V. Lacro, Lynn A. Sleeper, Mario Stylianou, Lynn Mahony, Gail D. Pearson, Maarten Groenink, Barbara J. Mulder, Aeilko H. Zwinderman, Julie De Backer, Anne M. De Paepe, Eloisa Arbustini, Guliz Erdem, Xu Yu Jin, Marcus D. Flather, Michael J. Mullen, Anne H. Child, Alberto Forteza, Arturo Evangelista, Hsin-Hui Chiu, Mei-Hwan Wu, George Sandor, Ami B. Bhatt, Mark A. Creager, Richard B. Devereux, Bart Loeys, J. Colin Forfar, Stefan Neubauer, Hugh Watkins, Catherine Boileau, Guillaume Jondeau, Harry C. Dietz, Colin Baigent

**Affiliations:** a Division of Cardiovascular Medicine, Radcliffe Department of Medicine, University of Oxford, Oxford, UK; b Clinical Trial Service Unit & Epidemiological Studies Unit, University of Oxford, Oxford, UK; c Children's Hospital Boston and Harvard Medical School, Boston, MA; d New England Research Institutes, Watertown, MA; e National Heart, Lung, and Blood Institute, NIH, Bethesda, MD; f University of Texas Southwestern Medical Center, Dallas, TX; g Academic Medical Center, University of Amsterdam, Amsterdam, The Netherlands; h Center for Medical Genetics and Department of Cardiology, University Hospital Ghent, Ghent, Belgium; i Center for Medical Genetics, University Hospital Ghent, Ghent, Belgium; j Centre for Inherited Cardiovascular Diseases, IRCCS Foundation, San Matteo Hospital, Pavia, Italy; k Clinical Trials and Evaluation Unit, Royal Brompton & Harefield NHS Trust, London, UK; l John Radcliffe Hospital, Oxford, UK; m Faculty of Medicine and Health Sciences, University of East Anglia, Norwich, UK; n UCL Institute of Cardiovascular Science, The Heart Hospital, London, UK; o Department of Cardiac and Vascular Sciences, St George's Hospital, University of London, London, UK; p Hospital Universitario 12 de Octubre, Madrid, Spain; q Marfan Sydrome Unit, Department of Cardiology, Hospital Vall d'Hebron, Barcelona, Spain; r Department of Pediatrics and Adult Congenital Heart Center, Taipei Medical University Hospital, Taipei, Taiwan; s Department of Pediatrics and Adult Congenital Heart Center, National Taiwan University Hospital, Taipei, Taiwan; t Children's Heart Centre, British Columbia's Children's Hospital, Vancouver, British Columbia, Canada; u Massachusetts General Hospital, Boston, MA; v Brigham and Women's Hospital and Harvard Medical School, Boston, MA; w Weill Cornell Medical College and New York-Presbyterian Hospital, New York, NY; x Center for Medical Genetics, Faculty of Medicine and Health Sciences, University of Antwerp and Antwerp University Hospital, Antwerp, Belgium; y Oxford Centre for Clinical Magnetic Resonance Research, Division of Cardiovascular Medicine, Radcliffe Department of Medicine, University of Oxford, Oxford, UK; z Radcliffe Department of Medicine, University of Oxford, Oxford, UK; aa Inserm LVTS U1148, Departement de Génétique, Hôpital Xavier Bichat-Claude Bernard, Paris, France; ab Centre National de Référence pour le syndrome de Marfan et apparentés, INSERM LVTS U1148, Service de Cardiologie, AP-HP Hôpital Bichat, Paris, France; ac McKusick-Nathans Institute of Genetic Medicine, Johns Hopkins University School of Medicine, and the Howard Hughes Medical Institute, Baltimore, MD

## Abstract

**Rationale:**

A number of randomized trials are underway, which will address the effects of angiotensin receptor blockers (ARBs) on aortic root enlargement and a range of other end points in patients with Marfan syndrome. If individual participant data from these trials were to be combined, a meta-analysis of the resulting data, totaling approximately 2,300 patients, would allow estimation across a number of trials of the treatment effects both of ARB therapy and of β-blockade.

Such an analysis would also allow estimation of treatment effects in particular subgroups of patients on a range of end points of interest and would allow a more powerful estimate of the effects of these treatments on a composite end point of several clinical outcomes than would be available from any individual trial.

**Design:**

A prospective, collaborative meta-analysis based on individual patient data from all randomized trials in Marfan syndrome of (i) ARBs versus placebo (or open-label control) and (ii) ARBs versus β-blockers will be performed.

A prospective study design, in which the principal hypotheses, trial eligibility criteria, analyses, and methods are specified in advance of the unblinding of the component trials, will help to limit bias owing to data-dependent emphasis on the results of particular trials. The use of individual patient data will allow for analysis of the effects of ARBs in particular patient subgroups and for time-to-event analysis for clinical outcomes.

The meta-analysis protocol summarized in this report was written on behalf of the Marfan Treatment Trialists' Collaboration and finalized in late 2012, without foreknowledge of the results of any component trial, and will be made available online (http://www.ctsu.ox.ac.uk/research/meta-trials).

## Background

Marfan syndrome is a heritable disorder of connective tissue, which typically produces symptoms and physical or imaging signs in several organ systems, with involvement of the cardiovascular, ocular, and musculoskeletal systems being particularly prominent.^1-5^ The most clinically important feature for most patients is a very high prevalence of aortic aneurysm formation (particularly, but not exclusively, in the aortic root at the sinuses of Valsalva), conferring a dramatically increased risk of potentially life-threatening aortic dissection and rupture.^6^

The prevalence of Marfan syndrome (based on hospital attendances) has recently been estimated at around 1 in 10,000 people,^7^ but the condition often goes undiagnosed, and most authorities accept a somewhat higher true prevalence of between 1 in 3,000^8^ and 1 in 5,000 people.^9^

Current medical therapy for Marfan syndrome consists mainly of β-blocker therapy. This approach, suggested by Halpern et al,^10^ was intended to reduce the rate of rise of aortic pressure, dP/dt, to reduce the forces imposed upon the aortic wall and so to reduce the rate of aortic dilation. The randomized evidence supporting the use of β-blockers is limited to a single, small, open-label trial.^11^ Many patients experience progressive aortic dilation despite such therapy,^12,13^ and prophylactic aortic root surgery is often required.

Remarkable progress has been made in recent years in understanding the pathophysiological basis of Marfan syndrome.^14^ Mutations in the *FBN-1* gene, which encodes the extracellular matrix protein, fibrillin-1, were identified as the cause of Marfan syndrome in a number of families,^15^ and modern DNA sequencing techniques can now identify *FBN-1* mutations in up to 92% of patients.^16^

Data from mouse models suggest that many of the manifestations of Marfan syndrome arise as a consequence of dysregulated Transforming Growth Factor β (TGFβ) signaling. The angiotensin receptor blocker (ARB) losartan (which down-regulates TGFβ signaling) rescued the aortic phenotype in a mouse model of Marfan syndrome,^17^ suggesting that targeted manipulation of TGFβ or its downstream pathways and regulators could prove to be productive strategies for the prevention of aortic disease in Marfan syndrome in patients.^18-29^

Several observational studies of ARBs in patients with Marfan syndrome have shown promising reductions in the rate of aortic dilation, both in combination with β-blockers^12,30^ and as monotherapy.^31^ Furthermore, 2 relatively small, randomized studies found reduced rates of aortic dilation in patients with Marfan syndrome (already taking β-blockers in most or all cases), randomly allocated to losartan, compared to patients who were randomly allocated to open-label control.^23,27^

### Rationale for a meta-analysis of ARB trials in Marfan syndrome

Ten randomized controlled trials of ARBs in patients with Marfan syndrome were in progress at the time of protocol writing, each designed to evaluate the effects of ARBs, compared to either β-blockade or placebo/open-label control, on aortic root size (and other aspects of cardiovascular and noncardiovascular structure and function).^18-29^ These studies plan to answer a number of important questions regarding the efficacy, safety, and tolerability of ARB therapy in different circumstances. Individually, however, some of these studies may not be large enough to answer reliably a number of important outstanding questions regarding the effects of ARB therapy in Marfan syndrome.

Meta-analyses of randomized trials can, by reducing random errors and tending to minimize biases, provide more reliable estimates of the effects of a particular treatment strategy than any individual study.^32^ If individual participant data from these trials of ARBs in Marfan syndrome were to be combined, a meta-analysis of the resulting data (from approximately 2,300 patients) would provide more precise estimates across a number of trials of the treatment effects both of ARB therapy and β-blockade. In particular, it will increase statistical power to address the question of whether the treatments studied influence a clinically important composite end point of several clinical outcomes.

A meta-analysis of all trials will—because of its large size—allow sources of variation in the effect of treatment (eg, by age, baseline aortic root dimensions, or genotype) to be explored. Finally, a meta-analysis may be able to explore subsidiary hypotheses about the effects of treatments on a range of outcomes of interest beyond aortic root dimensions.

The principal investigators of these trials were contacted in 2012 and were asked if they might be willing to join a collaborative group—the Marfan Treatment Trialists' Collaboration (MTTC)—in which it would be prospectively agreed that individual patient data from each trial would, after the completion of each trial, be provided to a central repository to allow for an individual patient data meta-analysis to be performed.

A prospective study design, in which the principal hypotheses, trial eligibility criteria, analyses, and methods are specified in advance of the unblinding of the component trials, will help to limit bias owing to data-dependent emphasis on the results of particular trials.

Members of the MTTC attended a series of meetings in Chicago in July 2012 and in Munich in August 2012 and subsequently agreed a protocol for such a meta-analysis in late 2012. Since the protocol was finalized, the results from 2 open-label trials have been published,^23,27^ and the results of the remaining trials are awaited.

This report summarizes the protocol and statistical analysis plan, which together define the rationale, trial eligibility criteria, analyses to be performed, and statistical methods that were agreed upon by members of the MTTC, without foreknowledge of the results of the component trials. The full protocol and detailed statistical analysis plan will be made available online (http://www.ctsu.ox.ac.uk/research/meta-trials).

## Aims

### Primary aim

The primary aim of this meta-analysis is to estimate the effect of (i) ARB therapy and (ii) β-blocker therapy, on change in aortic root size across a number of trials conducted in patients with Marfan syndrome and no prior aortic root surgery.

### Secondary aims

The secondary aims of this meta-analysis are to assess the effects of these 2 treatment modalities on:

1. change in aortic root size at the sinuses of Valsalva among different patient subgroups, defined on the basis of baseline characteristics (Table II);
2. cardiovascular outcomes, including a composite of aortic dissection, aortic root surgery, or death, and on individual components of this end point (Table III); and
3. clinically or biologically important secondary outcomes of interest (Table III).

## Methods

### Study eligibility and identification

Randomized trials in patients with Marfan syndrome (defined according to the 1996 Ghent criteria^33^ or the updated 2010 Ghent criteria^34^) are eligible for inclusion in the meta-analysis if they include a properly randomized comparison of one or both of (i) ARB versus placebo (or open-label control) or (ii) ARB versus β-blockers.

The inclusion of trials that have allocated patients to ARB versus placebo (or open-label control) and those that have allocated patients to ARB versus β-blockers allows not only for unbiased assessments of treatment effects of ARBs but also, indirectly, for an assessment of the effect of β-blockers versus placebo or open-label control.

Relevant trials were identified by searching online trial registries (eg, clinicaltrials.gov, ISRCTN, PUBMED); computer-aided and manual searches of journals; scrutiny of published trial protocols, abstracts and meeting proceedings, and the reference lists of review articles; and inquiry among colleagues, collaborating trialists, and manufacturers of ARBs. These sources will be rechecked periodically to identify trials that may be relevant but were not registered at the time of protocol drafting. The list of the trials currently identified as being eligible is shown in Table I.

### Analytic approach

The primary analytic approach for the meta-analysis will be according to the intention-to-treat principle, classifying each randomized subject according to their assigned treatment. Where participants discontinue allocated treatment strategies after randomization, data from such participants will continue to be included where available.

### Primary analysis

#### Primary outcome

The primary outcome will be the annual rate of change of body surface area (BSA)–adjusted aortic root dimension z-score, measured at the aortic sinuses of Valsalva. The z-score is a dimensionless quantity, representing the signed number of standard deviations (SDs) away from the mean where an observation lies.

For the primary analysis, data from subjects with prior aortic root surgery at enrollment (enrolled in only a minority of trials) will be excluded. For subjects who underwent aortic root surgery or who died during the follow-up period, measurements of aortic root dimensions obtained before aortic surgery or death will be included in the analysis. Measurements of aortic root dimensions obtained after aortic root surgery will not be included.

For each trial, the imaging method of estimating aortic root dimension used for that trial's primary analysis will be used as the primary outcome measure in this meta-analysis. Aortic root dimensions will be scaled for body size and normalized to appropriate reference populations. For each patient, linear slopes of rate of change of BSA-adjusted aortic root dimension z-score will be calculated.

Sources of variation in the method of aortic root measurement (including, for example, leading-edge to leading-edge method, compared to inner-edge to inner-edge method; systolic compared to diastolic measures) and in the methods used for body size indexation and reference population normalization will be explored and reported (see Secondary outcomes below).

#### Effect of ARB versus placebo and of ARB versus β-blocker on aortic root size

Between-trial variation in age-range, other eligibility criteria, and treatment protocol, a common analysis plan will be implemented separately within each participating trial. For each trial, the difference in mean annual rate of change of BSA-adjusted aortic root dimension z-score between patients allocated to ARBs and patients allocated to control therapy (ie, placebo/open-label control or β-blocker, depending on the trial) will be estimated, as will the standard error of this mean difference.

Standard inverse-variance–weighted methods for meta-analysis will then be used to estimate (separately) the effect on aortic root dimension of allocation to ARB versus placebo (or open-label control) (in the 6 trials that provide such a comparison) and the effect of allocation to ARB versus β-blocker (in the 5 trials that provide such a comparison).

Results will be considered to be statistically significant if the 2-sided *P* value is <.05, but chief emphasis will always be placed on the effect size estimate and its associated confidence intervals, which are the primary results of meta-analyses.

#### Effect of β-blockers on aortic root size: combining direct and indirect evidence

Only 1 of the 10 trials identified allows for a direct randomized assessment to be made of the effect of allocation to β-blockers (compared with control) on aortic root dimension (the Italian trial; Table I). However, an indirect assessment of the effect of β-blockers on aortic root dimension (and some other outcome measures) can be made from the other 9 trials, by combining the results from the 5 trials that only compared ARB versus β-blockers with the results from the 4 trials that only compared ARB versus placebo (or open-label control).

Specifically, if d_1_ (with variance v_1_) is the difference in mean annual rate of change in aortic root dimension estimated from the 5 trials that only compared ARB versus β-blockers and d_2_ (with variance v_2_) is the difference in mean annual rate of change in aortic root dimension estimated from the 4 trials that only compared ARB versus placebo/open control, then an indirect estimate of the effect of β-blockers is provided by d_2_− d_1_ (which has variance equal to v_1_ + v_2_).

The overall estimate of the effect on aortic root dimension of allocation to β-blocker is then provided by the inverse-variance–weighted average of the direct result from the Italian trial and the indirect evidence from the 9 other trials.^35^

For the comparison of ARB versus placebo, the randomization of >1200 patients should provide >90% power (with a 2-sided α = .05) to detect a 0.2-SD difference in annual rate of change of BSA-adjusted aortic root dimension z-score, whereas the randomization of >900 patients to ARB versus β-blocker would provide >80% power (with a 2-sided α = .05) to detect a between-group difference of 0.2 SD and >90% power to detect a 0.25-SD difference.

### Secondary analyses

#### Subgroup analyses

The availability of individual patient data will allow exploration of whether the benefits or hazards of treatment with ARBs or β-blockers are particularly great in certain types of patient, defined on the basis of characteristics present at the time of randomization.

The absolute effects on the primary outcome of allocation to ARBs (and, indirectly, of allocation to β-blockers) versus control will, therefore, be examined separately, according to a limited number of subgroups defined by prespecified baseline characteristics (Table II).

Tests for differences in the absolute treatment effect according to each characteristic (ie, tests for interaction) will be derived from standard tests for heterogeneity/trend across subgroups or, where needed, by fitting appropriate interaction terms with treatment allocation in a linear regression model.

Because the likelihood of a false-positive result (ie, a type I error) increases with the number of subgroup analyses performed, these tests will not be considered statistically significant unless the interaction *P* value is <.01 (and, even then, may be considered only as “hypothesis-generating”). Secondary analyses will always be identified as such in manuscripts arising from this work.

Some baseline characteristics (eg, sex, age at enrollment) are expected to be available for all enrolled participants in all trials. Other baseline characteristics (eg, markers of arterial elasticity) may only be available from a subset of trials. In these cases, analyses will be performed on the available data, where sufficient data are available for reliable and informative analysis. Analyses will be performed first on all trials evaluating the ARB losartan and, subsequently, on all ARB trials, irrespective of the ARB used.

Although data from subjects with a history of aortic root surgery at enrollment (enrolled in only a minority of trials) will be excluded from the primary analysis, a secondary analysis will be performed using data exclusively from those subjects who gave a history of aortic root surgery at baseline. For this analysis, absolute ascending aortic root dimensions will be the main outcome of interest, and aortic arch dimensions and descending aortic dimensions will be considered to be the principal secondary outcome measures for this analysis.

For each trial, we will report the proportion of subjects in whom genetic testing has been performed, the proportion of subjects in whom mutations have been identified, and the loci affected. Subgroup analyses will be performed based on presence or absence of mutations at particular loci (eg, *FBN-1* mutations) and, where possible, based on the predicted functional consequence of particular categories of mutation (eg, nonsense vs missense *FBN-1* mutations) among those subjects with identified mutations.

Circulating biomarkers of vascular function (eg, TGFβ; matrix metalloproteinases; and their circulating regulators, fibrillin-1 fragments) or samples suitable for analyses of these and other biomarkers may be available from some trials. Analyses of these biomarkers may be feasible, but such analyses will only be considered to be hypothesis generating.

#### Secondary outcomes

Analyses will be performed to estimate the effect of ARB therapy (and, indirectly, of β-blockers) versus control on certain prespecified secondary outcome measures (Table III).

Continuous measures will be analyzed by calculating the inverse-variance–weighted average result across the trials (using, when appropriate, repeated-measures methods for each trial). Time-to-event outcomes will be analyzed using log-rank methods for meta-analyses. All *P* values will be 2 sided, with a significance level of .05 being deemed significant, but any marginally significant results will be interpreted with appropriate caution and may be considered only as hypothesis generating.

Differences in the proportional effects of allocation to ARB (and, indirectly, of allocation to β-blocker) on the prespecified secondary outcomes by baseline characteristics (Table II) will also be assessed.

A sensitivity analysis will also estimate the effects of different methods of BSA estimation, body size estimation (height, BSA, BSA^0.5^), and normalization on the primary outcome and on secondary outcomes where appropriate.

Exploratory analyses will be conducted to assess the sensitivity of the final results to the analysis method used. In particular, the effect size estimates for each of the outcomes will be compared to those yielded by “random-effects” models, and if these are substantially discrepant, the final report will include a discussion of possible reasons for any differences.

### Interim analyses

The final analysis will seek to include all trials of ARBs worldwide. Interim analyses may be performed if ≥1 trials are delayed, and these analyses may be submitted for publication before the completion of all trials. A separate analysis, confined to those trials evaluating the ARB losartan, will be performed before an analysis of trials including all ARBs.

### Collaborative group structure, organization, and management

The meta-analysis will be undertaken by the MTTC, which is a collaborative group of trialists, meta-analysts, statisticians, biological scientists, and clinicians. The group includes representatives of each collaborating trial and meets periodically to discuss the design, conduct, and reporting of this meta-analysis. The study is supported and coordinated by a secretariat based at the Clinical Trial Service Unit & Epidemiological Studies Unit, University of Oxford, UK, and at the Division of Cardiovascular Medicine, Radcliffe Department of Medicine, University of Oxford, UK.

### Protocol preparation and publication policy

Draft versions of the protocol and statistical analysis plan were prepared by the secretariat in spring 2012 and were reviewed at the meetings of the MTTC in Chicago in July 2012 and in Munich in August 2012. Drafts were circulated to all members of the collaboration throughout the process, and the documents were finalized after a series of teleconferences in late 2012.

Manuscripts arising from the meta-analysis will be prepared by the secretariat and reviewed by the collaborative group. The main findings of the meta-analysis will be submitted for publication in a peer-reviewed journal irrespective of the outcome of the overview. All publications will be submitted in the name of the MTTC, listing all collaboration members. Further information regarding the approach to analysis is available on request from the corresponding author, and inquiries from those wishing to perform similar types of analyses would be particularly welcome.

## Disclosures

The study is supported by funding from The Marfan Foundation; the Medical Research Council; and the British Heart Foundation Centre for Research Excellence, Oxford. Alex Pitcher acknowledges funding support from the Marfan Foundation; the Gibson fund; the NIHR Biomedical Research Centre, Oxford; and the British Heart Foundation Centre for Research Excellence (grant code RE/13/1/30181). Alex Pitcher is supported by the Academy of Medical Sciences Clinical Lecturer Starter Grant scheme, which is administered by the Academy on behalf of the the Academy, the Wellcome Trust, the Medical Research Council, the British Heart Foundation, Arthritis Research UK, Prostate Cancer UK and the Royal College of Physicians. Eloisa Arbustini acknowledges Telethon grant number GGP08238.

## Declaration

The authors declare that they have no conflicts of interest. All authors contributed to the design of the meta-analysis described in this work and have reviewed and approved the final manuscript.

## Figures and Tables

**Table I:** Register of all current and planned randomized trials of angiotensin receptor antagonists in Marfan syndrome

Study	Sample size	Diagnostic criteria	Aortic size criteria	Age (y)	Treatment	Comparator	Follow-up duration (mo)	Primary end point	Timing of follow-up visits (mo)	Imaging methods	Study end date
ARB vs β-blocker											
US (Pediatric Heart Network)	608	Ghent	z-score >3.0 and aortic root ≤5.0 cm	0.5-25	Losartan 0.4-1.4 mg/kg daily	Atenolol	36	Rate of change in aortic root (sinus of Valsalva) BSA-adjusted z-score	6, 12, 24, 36	Echo	Nov 14
Italy (ARB vs β-blocker arms)	156	Ghent and mutation in FBN1	z-score ≥2 or aortic root >3.8 cm (F)/>4.0 cm (M) and <5.0 cm	1-55	Losartan target dose 100 mg daily	Nebivolol	48	Aortic root growth rate	12, 24, 36, 48	Echo	Sept 15
Spain	150	Ghent	No minimum, aortic root ≤4.5 cm	5-60	Losartan 12.5-100 mg daily	Atenolol	36	Progression of aortic dilation	6, 12, 24, 36	CMR	Sept 14
US (Boston)	50	Ghent	Unrestricted	25+	Losartan	Atenolol	6	Arterial stiffness measures	6	Echo	Sept 14
Canada	17	Ghent	Unrestricted	12-25	Losartan	Atenolol	12	Pulse wave velocity	12	Echo	Sept 14

ARB vs placebo (or open-label control)											
UK	490	Revised Ghent	z-score >0, aortic root <4.5 cm	≥6-40	Irbesartan 150-300 mg daily⁎	Placebo⁎	48-60	Absolute change in aortic root diameter per year	12, 24, 36, 48, 60	Echo	Sept 18
The Netherlands	233	Ghent	No minimum size, but aortic root <5 cm	≥18	Losartan 50-100 mg daily⁎	Open-label control⁎	36	Largest change at any aortic level by MRI from baseline to end of study	12, 24, 36	CMR (0 and 36)	Nov 13
France	300	Ghent	Unrestricted	≥10	Losartan 50-100 mg daily⁎	Placebo⁎	36	Rate of change of normalized aortic root diameter expressed as z-score	6, 12, 18, 24, 30, 36	Echo	Sept 14
Italy (ARB + β-blocker vs β-blocker arms)	156	Ghent and mutation in FBN1	z-score ≥2 or aortic root >3.8 cm (F)/>4.0 cm (M) and <5.0 cm	1-55	Losartan 100 mg and nebivolol	Nebivolol	48	Aortic root growth rate	12, 24, 36, 48	Echo	Sept 15
Belgium	39	Revised Ghent	z-score ≥2.0	≥10	Losartan 25-100 mg daily⁎	Placebo⁎	36	Rate of change in the aortic root by linear regression of the z-score	6, 12, 24, 36	Echo (primary) and CMR (0 and 36)	Dec 14
Taiwan	29	Ghent	Recognized aortic dilation	1+	Losartan and either atenolol or propranolol	Atenolol or propranolol	35	Aortic root growth rate	35	Echo	Mar 13

Imaging methods: where >1 imaging method is listed (primary) indicates the method used for the primary outcome. Abbreviations: *MRI*, magnetic resonance imaging; *CMR*, cardiovascular magnetic resonance

⁎ Trials based in the UK, France, Belgium, and the Netherlands allow enrolled patients to remain on their baseline therapy (usually, but not always β-blockers). The Italian and Taiwanese trials mandate β-blocker in the comparator arm(s). The Italian trial is randomizing 235 subjects in a 3-way, 1:1:1 randomization, to losartan alone, nebivolol alone, or losartan + nebivolol. Sample per arm is estimated as 78 (235/3) and 156 per comparison (78 × 2).

**Table II:** Patient subgroup definitions based on characteristics at randomization

• Age at randomization⁎ • Sex • Family history of aortic dissection in a first-degree family member (i) at any age and (ii) occurring in the family member at an age ≤40 y • The presence or absence of: ▪ ectopia lentis ▪ dural ectasia ▪ musculoskeletal involvement† • Aortic root dimension at the sinuses of Valsalva at enrollment, indexed to body surface area and expressed as a z-score‡ • Baseline age-adjusted imaging markers of arterial stiffness and elasticity • Presence and type of variants in genes encoding components of the extracellular matrix including fibrillin-1; the TGFβ pathway; the renin-angiotensin system; the β-adrenergic system; and pathways involved in the binding, transport, metabolism, or excretion of ARBs and/or β-blockers • Circulating biomarkers of vascular function (including TGFβ level where available) at baseline • Use of β-blockers at baseline • Use of calcium-channel blockers at baseline • Use of HMG CoA reductase inhibitors (statins) at baseline • Prior history of aortic root surgery at baseline • Systolic and diastolic blood pressure at baseline as a continuous measure§

⁎ Both the linear dependence of the absolute treatment effect on age and the separate effect of treatment allocation among patients aged ≤16 years versus >16 years will be assessed.

† Defined as >4 major manifestations reaching a major criterion in the 1996 nosology.

‡ As a continuous measure and categorized as <4.5 versus ≥4.5.

§ As a continuous measure and categorized as <140 versus >140 systolic and <90 versus >90 diastolic.

**Table III:** Secondary endpoints and outcome measures

• The composite end point of aortic dissection, aortic root surgery, or death⁎ • Annual rate of change in absolute aortic dimensions at the sinuses of Valsalva† • Annual rate of change in absolute aortic dimensions at the ascending aorta† • Annual rate of change in absolute aortic dimensions at other aortic sites†‡ • Annual rate of change in BSA-adjusted, normalized aortic dimensions, expressed as a z-score†‡ • Annual rate of change in proportional aortic root size‡ • Annual rate of change in absolute and BSA-adjusted dimensions of the pulmonary artery • The incidence of moderate to severe aortic valve regurgitation • The incidence of moderate to severe mitral valve regurgitation • The incidence of aortic valve–sparing aortic root surgery and combined aortic valve and aortic root replacement • Annual rate of change of measures of left ventricular cavity size, wall thickness, and systolic function§ • Annual rate of change of brachial systolic, diastolic, and mean arterial pressure and pulse pressure • Rate of change of age-adjusted measures of arterial stiffness/elasticity • Rate of change of levels of circulating biomarkers of vascular function (including TGFβ where available) • Annual rate of change of markers of somatic growth and disproportion║ • Tolerability and side effects of therapy, frequency, and nature of adverse drug reactions and quality-of-life indices and the proportion of treatment failures, discontinuations, and/or patient drop-outs.

⁎ And of each of these components separately.

† Sensitivity analysis will also estimate the effects of different imaging methods (systole vs diastole; inner-edge to inner-edge vs leading-edge to leading-edge method, echo versus magnetic resonance imaging).

‡ At the aortic annulus, sinuses of Valsalva, sinotubular junction, ascending aorta, aortic arch, and descending aorta.

§ End-diastolic dimension, end-diastolic volume, end-systolic dimension, end-systolic volume, left ventricular wall thickness (septum), left ventricular wall thickness (posterior wall), left ventricular mass, left ventricular mass/volume ratio, fractional shortening, and ejection fraction, each indexed to body size and normalized where appropriate.

║ Height, weight, body surface area, body mass index z-scores indexed to age, and markers of skeletal disproportion (arm span-to-height ratio and upper-segment-to-lower-segment ratio) with age at enrollment and height at enrollment as covariates.
